# Constitutive asymmetric dimerization drives oncogenic activation of epidermal growth factor receptor carboxyl-terminal deletion mutants

**DOI:** 10.18632/oncotarget.3559

**Published:** 2015-03-12

**Authors:** Angela K.J. Park, Joshua M. Francis, Woong-Yang Park, Joon-Oh Park, Jeonghee Cho

**Affiliations:** ^1^ Samsung Genome Institute, Samsung Medical Center, Seoul, Republic of Korea; ^2^ Samsung Advanced Institute for Health Sciences and Technology, SungKyunKwan University, Seoul, Republic of Korea; ^3^ The Broad Institute of Harvard and MIT, Cambridge, Massachusetts, USA; ^4^ Division of Hematology-Oncology, Samsung Medical Center, Sungkyunkwan University School of Medicine, Seoul, Republic of Korea

**Keywords:** epidermal growth factor receptor, EGFR targeted therapy, asymmetric dimerization, EGFR C-terminal domain deletion

## Abstract

Genomic alterations targeting the Epidermal Growth Factor Receptor (*EGFR*) gene have been strongly associated with cancer pathogenesis. The clinical effectiveness of EGFR targeted therapies, including small molecules directed against the kinase domain such as gefitinib, erlotinib and afatinib, have been proven successful in treating non-small cell lung cancer patients with tumors harboring EGFR kinase domain mutations. Recent large-scale genomic studies in glioblastoma and lung cancer have identified an additional class of oncogenic mutations caused by the intragenic deletion of carboxy-terminal coding regions. Here, we report that combinations of exonic deletions of exon 25 to 28 lead to the oncogenic activation of EGF receptor in the absence of ligand and consequent cellular transformation, indicating a significant role of C-terminal domain in modulating EGFR activation. Furthermore, we show that the oncogenic activity of the resulting C-terminal deletion mutants are efficiently inhibited by EGFR-targeted drugs including erlotinib, afatinib, dacomitinib as well as cetuximab, expanding the therapeutic rationale of cancer genome-based EGFR targeted approaches. Finally, *in vivo* and *in vitro* preclinical studies demonstrate that constitutive asymmetric dimerization in mutant EGFR is a key mechanism for oncogenic activation and tumorigenesis by C-terminal deletion mutants. Therefore, our data provide compelling evidence for oncogenic activation of C-terminal deletion mutants at the molecular level and we propose that C-terminal deletion status of EGFR can be considered as a potential genomic marker for EGFR-targeted therapy.

## INTRODUCTION

Aberrant activation of Epidermal Growth Factor Receptor (EGFR), a member of the ErbB family of receptor tyrosine kinases, plays a central role in development and progression of cancer [1, 2]. Different classes of genomic alterations involving *EGFR* identified in cancer, including lung adenocarcinoma and glioblastoma (GBM), have been shown to be responsible for altered EGFR regulation and cellular transformation [3, 4]. In lung adenocarcinoma, the most frequent somatic mutations occur within the kinase domain of EGFR, including L858R in exon 21 and small in-frame deletions in exon 19 [5, 6]. Importantly, these two particular somatic mutations are associated with a clinical response to the small molecule EGFR kinase inhibitors, such as gefitinib, erlotinib, and afatinib [7-11]. In contrast, *EGFR* alterations identified in GBM include intragenic deletions targeting exons 2 to 7 deletion (known as EGFR vIII), exons 14 to 15 (known as EGFRvII) and somatic mutations within the extracellular domain of EGFR, but kinase domain mutations are relatively rare [12-16]. Despite *in vitro* experiments demonstrating the effectiveness of small molecule inhibitors on GBM-specific oncogenic EGFR variants, they have not yielded consistent responses in GBM patients harboring such mutations [17, 18].

Recent large-scale genomic analyses identified intragenic deletion mutations within the EGFR carboxy-terminal domain in GBM and lung adenocarcinoma [16, 19-21]. Subsequent studies have shown that the resulting C-terminal truncation variants of EGFR have oncogenic potential to promote cellular transformation and tumorigenesis [16, 19, 21, 22]. Importantly, FDA-approved EGFR targeted drugs including erlotinib, and cetuximab, a humanized anti-EGFR monoclonal antibody, effectively inhibit the oncogenic activation of C-terminal deletion EGFR mutants, demonstrating that both drugs may be promising therapeutic agents in treating cancer patients harboring such deletion mutations [19, 23]. The second generation EGFR kinase inhibitors, such as FDA-approved afatinib and dacomitnib, which is currently in phase III trial, are being actively investigated as they have demonstrated better efficacy than erlotinib and shown to overcome EGFR gatekeeper mutation, T790M [24-26]. However, their efficacies against C-terminal deletion EGFR mutants have not been investigated yet.

Three-dimensional structural analysis of EGFR has revealed the importance of ligand-induced asymmetric dimerization mediated by the N-lobe and the C-lobe of the EGFR kinase domain in receptor activation [27-29]. This finding was further supported by functional evidence that disruption of asymmetric dimerization through substitution mutations at the dimerization interface, such as L704N (receiver-impairing mutation) in the N-lobe and I941R (activator-impairing mutation) in the C-lobe, impair ligand-induced EGFR activation and consequent cellular transformation [30]. Mouse tumors induced by dimerization-dependent L858R and G719S mutants respond dramatically to cetuximab, whereas tumors driven by dimerization-independent mutant exon 20 insertion mutant are resistant. Therefore, it was proposed that EGFR mutation status may be a predictive factor of clinical response to cetuximab as a close correlation exists between dimerization dependency and its pharmacological effects [30, 31].

Several genomic rearrangements leading to oncogenic C-terminal deletion mutant EGFR have been identified in cancer, however the molecular mechanisms mediating cellular transformation by these oncogenic mutants is unknown. For a comprehensive assessment of their biological role and clinical applications, we characterized a complete panel of both previously identified as well as not yet discovered *EGFR* C-terminal deletion mutations by establishing stable cell lines harboring multiple or single exon deletions within exon 25 to 28, thereby expressing 10 different EGFR C-terminal deletion variants. Subsequently, we examined the functional consequence of these deletions in regulating oncogenic activation of EGFR and sensitivity to EGFR targeted drugs. In particular, we sought to address whether asymmetric dimerization is required for cellular transformation through activation of GBM and lung cancer-derived oncogenic C-terminal deletion mutants. Our *in vivo* and *in vitro* preclinical studies demonstrate that C-terminal exonic deletion mutants are oncogenically active in the absence of ligand and sensitive to EGFR targeted therapies, and more importantly, that their oncogenic potential depends on the asymmetric dimerization of kinase domain.

## RESULTS

### EGFR CTED mutants have transformation potential

**Figure 1:**
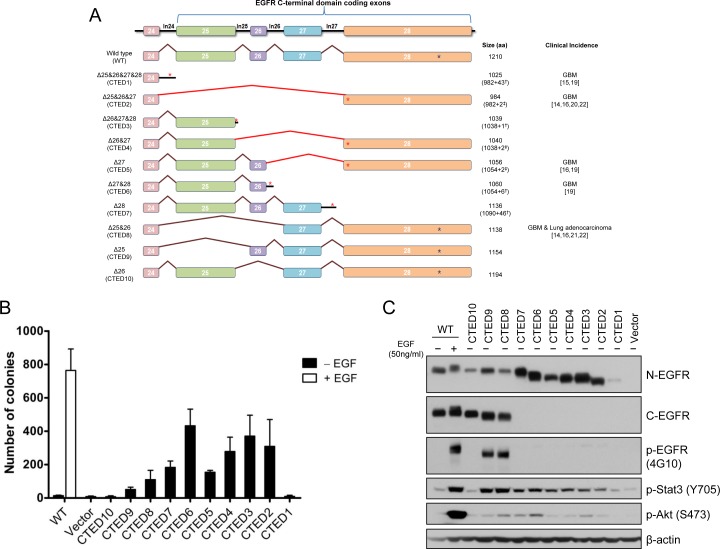
EGFR deletion variants resulting from EGFR C-terminal domain exonic deletion (CTED) mutation have transforming potential (A) Schematic diagram of *EGFR* C-terminal exonic deletion mutations characterized in this study. Brown lines indicate exons splicing events whereas red lines indicate out-of-frame splicing. The total translated amino acid numbers of each CTED mutant and clinical incidence of each genomic alteration are indicated. For CTED1, CTED3, CTED6, and CTED7, exonic deletion results in inclusion of intron due to the loss of splicing acceptor site. For CTED2, CTED4, and CTED5, exonic deletion generates a frameshifted exon 28, with the addition of Asn (N) and Thr (T) followed by early termination. The number of amino acids added by inclusion of intron or frameshift of exon 28 are indicated with (^†^) and (^‡^) respectively, followed by + sign. Red asterisk (*) indicate the predicted intron-encoded stop codon and stop codons generated by frameshift and blue asterisk (*) indicate location of the EGFR stop codon. (B) NIH-3T3 cells stably expressing the indicated CTED mutant or WT EGFR were assayed for anchorage-independent growth in soft agar in the presence or absence of EGF. The bar graph depicts the number of colonies formed in soft agar (n=3, mean + SD). (C) Cell lysates prepared from NIH-3T3 cells expressing the indicated EGFR CTED mutants or WT EGFR were subjected to immunoblotting with antibodies against N-terminal epitope EGFR, C-terminal epitope EGFR, phospho-tyrosine (4G10), phospho-Stat3, phospho-Stat5, phospho-Akt, and β-actin.

In order to systemically characterize the oncogenic potential of C-terminal deletion (CTED) mutants, we generated a series of EGFR expression constructs encoding the 10 possible combinations of exon 25 to 28 deletions as shown in Fig. 1A. The resulting EGFR deletion variants can be classified into 3 different subgroups (see the figure legends for detail); 1) out-of-frame deletion mutants lacking exon 25-28, exon 26-28, exon 27-28 or exon 28 (designated CTED1, CTED3, CTED6, and CTED7, respectively) with intron-encoded stop codon, 2) out-of-frame deletion mutants lacking exon 25-27 or exon 26-27 or exon 27 (designated CTED2, CTED4, and CTED5, respectively) with premature stop codon in subsequent exon and 3) in-frame deletion mutants lacking exon 25 or exon 25-26 or exon 26 (designated CTED8, CTED9, and CTED10, respectively). These deletion variants as well as wild-type (WT) EGFR were stably expressed in NIH-3T3 cells and the oncogenic phenotype of the resulting cell lines was assessed through anchorage-independent growth in soft agar. The oncogenic activity of cancer-derived CTED2, CTED5, and CTED8 mutants have been previously established and serve as positive controls in this study [19, 22].

NIH-3T3 cells expressing WT EGFR formed colonies in soft agar only upon the addition of EGF, while the CTED mutants, with the exception of CTED1 and CTED10, formed colonies in the absence of EGF (Fig. 1B). These results indicate that the deletion of exon 26 (1039-1054 aa) alone was not sufficient to induce cellular transformation of NIH-3T3 cells. In agreement with a previous report [22], we also noted a correlation between the deleted length of the C-terminal domain and the degree of transformation, suggesting that a minimal length or region is necessary to provide a growth advantage.

Taken together, these findings demonstrate that in addition to the previously characterized CTED1, CTED2, CTED5 and CTED8 mutants found in GBM and lung cancer, other EGFR truncated mutants produced by different intragenic deletions of C-terminal domain are able to induce cellular transformation in NIH-3T3 cells.

### EGFR CTED mutants constitutively activate downstream signaling

Next, we examined tyrosine phosphorylation on mutant EGFR as well as downstream signaling molecules including Stat3 and Akt to investigate whether cellular transformation induced by the EGFR CTED mutants is associated with ligand-independent activation of receptor. NIH3T3 cells have low endogenous levels of EGFR so phosphorylation signal represents exogenous mutants only. While tyrosine phosphorylation of WT EGFR was detected only upon EGF stimulation, CTED8 and CTED9 in-frame deletion mutants were constitutively phosphorylated and enzymatically active in the absence of EGF as evidenced by downstream phosphorylation of Stat3 (Fig. 1C). The phosphorylation status of CTED1 to CTED7 mutants was not readily detectable due to the absence of the tyrosine residues, however by using Stat3 and Akt as readouts, we observed constitutive activation of all mutants but CTED1 and CTED10, consistent with the absence of colonies in soft agar (Fig. 1C). In line with the varying oncogenic ability observed in the soft agar assay, although CTED2 to CTED7 mutants were constitutively activated, they exhibited varying ability to activate downstream molecules, as demonstrated by different extent of Akt phosphorylation. Overall, we found that the CTED2 to CTED9 mutants are oncogenic and consequently able to induce activation of downstream signaling pathways including Stat3 and Akt. These data are consistent with previous reports that C-terminal truncated mutants retain ability to transduce signal [22, 32, 33].

### EGFR CTED mutants exhibit sensitivity to EGFR inhibitors *in vitro* and *in vivo*

Previous studies have shown that the oncogenic activity of cancer-derived CTED2, CTED5 and CTED8 mutants was effectively suppressed by the EGFR inhibitors erlotinib or cetuximab both *in vitro* and *in vivo* [19, 21]. To further investigate the clinical utility of these anti-EGFR drugs, we profiled the panel of CTED mutants. First, Ba/F3 cells were transformed through constitutive expression of all CTED mutants leading to IL-3 independence while parental Ba/F3 cells failed to grow [34]. Of note, unlike the NIH-3T3 cells, ectopic expression of both CTED1 and CTED10 mutants were able to transform Ba/F3 cells (Fig. S1A). These data further confirmed the transforming potential of CTED mutants in a second cell line system. The difference of transforming ability of the CTED1 and CTED10 mutants in two cell lines is unclear, but we hypothesize that differential EGFR expression may be responsible for the observed discrepancy.

**Figure 2:**
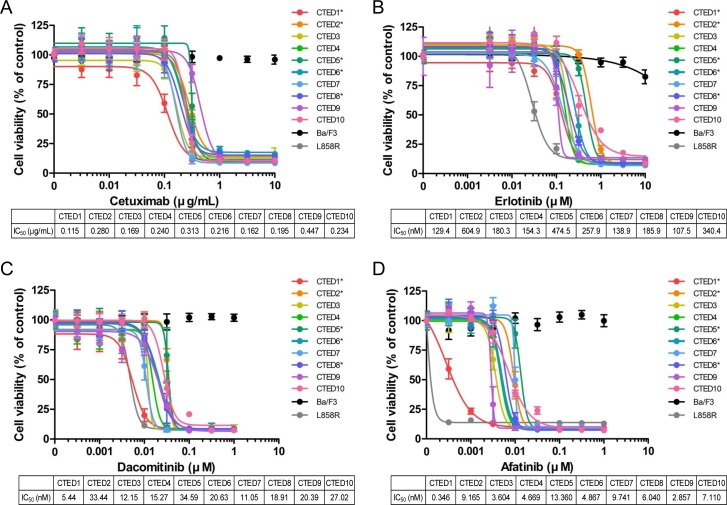
Oncogenic EGFR CTED mutants are sensitive to EGFR inhibitors *in vitro* Growth of Ba/F3 cells transformed by CTED1 to CTED10 mutants was suppressed by either cetuximab (A), erlotinib (B), dacomitinib (C), or afatinib (D). Ba/F3 cells transformed by indicated CTED mutants were treated with the four EGFR inhibitors at the concentrations indicated for 72 hours and assayed for cell viability using Cell Counting Kit-8 reagents. Parental and Ba/F3 cells carrying the L858R mutant of EGFR were used as controls. The results are presented as a mean ±SD of sextuplicate wells and are representative of three independent experiments. Asterisk (*) indicates previously reported CTED mutants in GBM and lung adenocarcinoma.

The CTED mutant transformed Ba/F3 cells were then tested for sensitivity to EGFR inhibitors, including cetuximab and erlotinib, as well as second generation irreversible EGFR/ERBB2 dual inhibitors afatinib and dacomitinib. All EGFR-targeted drugs effectively suppressed the growth of Ba/F3 cells expressing CTED mutants in a dose dependent manner (Fig. 2). The sensitivity of CTED2, CTED5 and CTED8 expressing cells to erlotinib is comparable with our previous report [19, 21]. In contrast, the same drugs showed no inhibitory effects on the parental Ba/F3 cells. In addition, we observed that the second generation EGFR inhibitors afatinib and dacomitinib were more potent, 100-fold and 10-fold respectively, at suppressing the growth of CTED mutants compared to the reversible inhibitor, erlotinib [25, 35]. Intriguingly, we found that cells expressing GBM-derived CTED1 mutant exhibited the highest sensitivity to three of the EGFR inhibitors (afatinib, dacomitinib, and cetuximab) whereas cells expressing GBM-derived CTED5 mutant exhibited the lowest sensitivity in two of the tested inhibitors (afatinib and dacomitinib). Other CTED mutants remained close to the mean IC_50_ values, which were ~0.237μg/ml, ~257.4nM, ~6.18nM, and ~19.9nM, for cetuximab, erlotinib, afatinib, and dacomitinib, respectively. Of note, we observed that lung cancer-derived L858R mutant responded more sensitively to erlotinib and afatinib than CTED mutants, but exhibited similar sensitivity to cetuximab and dacomitinib. Consistent with this result, these four drugs decreased constitutive phosphorylation of Stat5 and Akt in patient-derived CTED transformed Ba/F3 cells, but not in parental Ba/F3 cells, suggesting that the oncogenic activity of CTED mutants are specifically targeted and inhibited by these drugs (Fig. S1B-E).

**Figure 3:**
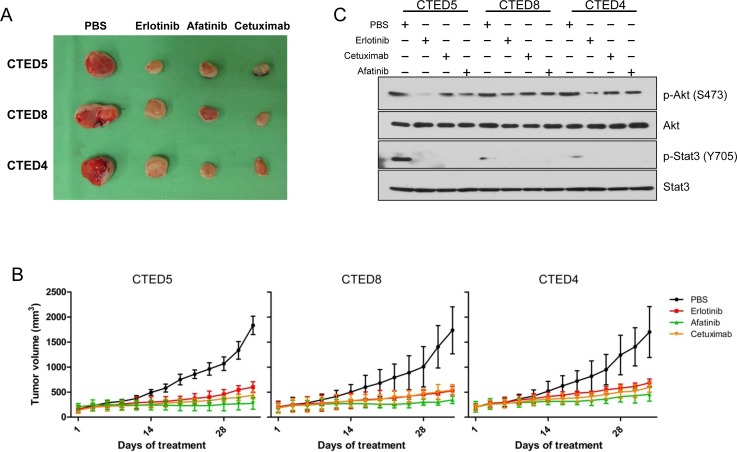
EGFR CTED mutant induced tumorigenesis is suppressed by either erlotinib, afatinib, or cetuximab (A) The images are representative of tumor resection from xenografted mice injected with NIH-3T3 cells expressing CTED5, CTED8, or CTED4 following approximately 5 weeks of EGFR inhibitors or PBS treatment. (B) The growth of mouse tumors driven by CTED5, CTED8, and CTED4 mutants was significantly suppressed by erlotinib, afatinib, or cetuximab. Fourteen days after cell injection, when tumors reached 50 to 70 mm^3^ in size, erlotinib, afatinib (50mg/kg, gavage), or cetuximab (50mg/kg, IP) was administered 3 times per week for 5 weeks. Tumor size was measured 3 times per week, and the volume was determined according to the formula V=ab^2^/2, where a and b were tumor length and width, respectively (n=12 for each treatment group, mean ± SD). IP, intraperitoneal. (C) Lysates prepared from the untreated or cetuximab, erlotinib, afatinib, or dacomitinib treated xenograft tumors were subjected to immunoblotting with total or phospho-specific antibodies against Akt, and Stat3.

To further explore the therapeutic efficacy of cetuximab, erlotinib, and afatinib *in vivo*, we expanded our studies to subcutaneous xenografts of NIH-3T3 cells stably expressing CTED4, CTED5 and CTED8 mutants. We chose CTED5 and CTED8 as a representative of various patient-derived oncogenic CTED mutants and CTED4 as a representative of hypothetical, or yet to be discovered CTED mutants. In line with our previous report [19], we found that treatment of the EGFR-targeted drugs dramatically suppressed tumor formation driven by CTED4, CTED5, and CTED8 as compared to PBS control (Fig. 3A and 3B). Furthermore, in line with *in vitro* results, constitutive phosphorylation of Stat3, but not Akt, was robustly diminished in the tumors treated with all tested drugs (Fig. 3C). These results demonstrate that in addition to the GBM and lung cancer-derived CTED 5 and CTED8 mutants, CTED4 mutant is also tumorigenic and that tumors derived from these mutants exhibit a significant response to cetuximab, erlotinib and afatinib.

### Oncogenic potential of EGFR CTED mutants depends on asymmetric dimerization

A subset of EGFR mutants, including L858R, G719S and G724S undergo constitutive asymmetric dimerization of the kinase domain contributing to the oncogenic activation of mutant EGFR [30, 31], demonstrating that dimer formation is an essential step required for cell proliferation by ErbB family members [36]. We sought to determine whether the same asymmetric dimerization-dependent activation mechanism is responsible for the oncogenic activity and consequent cellular transformation by the CTED mutants. EGFR expression constructs that combined receiver-impairing mutations (L704N or L760R) or activator-impairing mutations (I941R or M952R) with oncogenic CTED4, CTED5, and CTED8 mutants were stably expressed in NIH-3T3 cells and assayed for their ability to grow in soft agar. The transforming ability of dimerization-dependent mutants is predicted to be abolished by introduction of *cis* mutation of L704N, L760R, I941R, or M952R in CTED mutant [30, 31].

**Figure 4:**
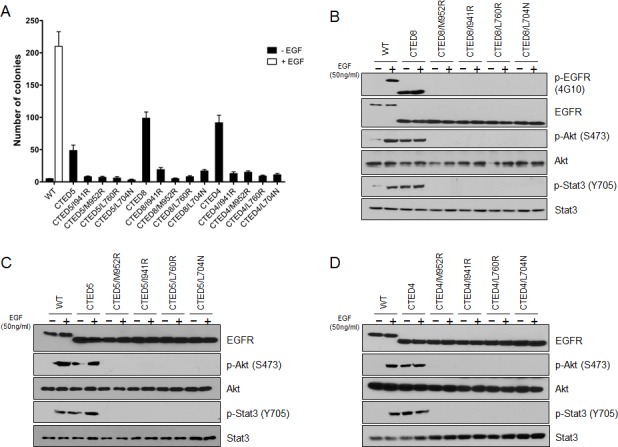
Disruption of asymmetric dimerization has substantial effects on the transforming activity of CTED mutants (A) NIH-3T3 cells stably expressing the indicated EGFR CTED mutants with or without receiver-impairing (L760R or L704N) or activator-impairing (I941R/M952R) mutations were assayed for anchorage-independent growth in soft agar. The bar graph depicts the number of colonies formed in soft agar (n=3, mean + SD). (B-D) NIH-3T3 cells expressing the indicated EGFR CTED mutants or WT EGFR were incubated for 5 min with or without EGF (50ng/ml), and the resulting lysates were subjected to immunoblotting with antibodies against total or phospho-specific EGFR, Stat3, and Akt.

We found that introduction of dimerization-impairing mutations into CTED4, CTED5 and CTED8 mutants led to substantial decreases in soft agar colony formation (Fig. 4A), demonstrating that these mutants are dependent on asymmetric dimerization for their oncogenic potential. These results are similar with what has been reported for the dimerization-dependent L858R, G719S and G724S mutants [30, 31]. Next, we assessed the biochemical consequences of disrupting asymmetric dimerization in oncogenic CTED mutants by studying the phosphorylation patterns of EGFR directly for CTED8 or indirectly for CTED4 and CTED5 through downstream signaling molecules (Fig. 4B-D). As suggested by the results of colony formation assay, no detectable EGFR, Stat3 and Akt phosphorylation was observed in all dimerization-impaired *cis* mutants of CTED4, CTED5 or CTED8. EGF treatment was not able to restore phosphorylation in these dimerization-impaired mutants, further confirming that the oncogenic activity of CTED4, CTED5 or CTED8 mutants require asymmetric dimerization.

**Figure 5:**
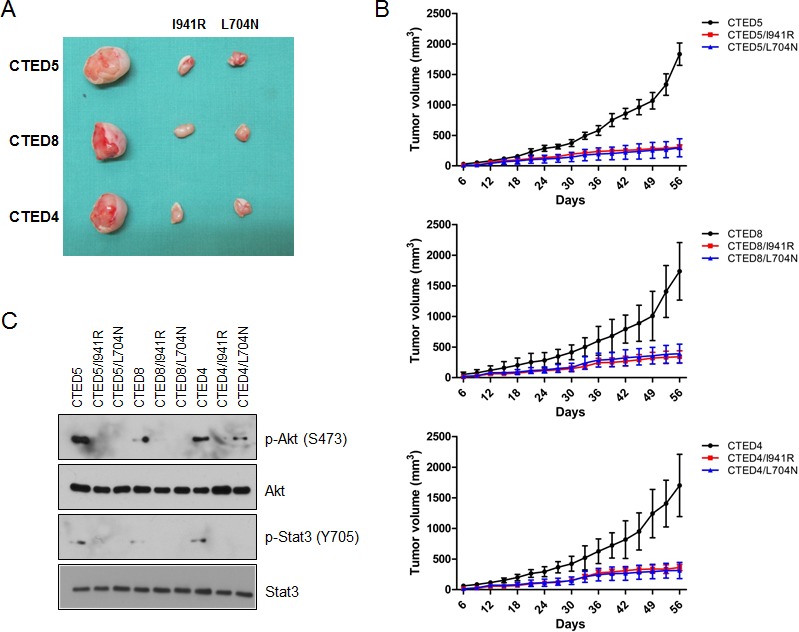
Dimerization impaired CTED mutants fail to induce tumorigenesis in xenograft mouse model (A) The images are representative of tumor resection from xenografted mice harboring NIH-3T3 cells stably expressing either CTED4, CTED5, or CTED8 with or without receiver mutation (I941R) or activator mutation (L704N). (B) The growth of mouse tumors driven by CTED4, CTED5, and CTED8 mutants were significantly suppressed by dimerization impairing L704N and I941R mutations. Tumor size was measured 3 times per week, and the volume was determined according to the formula V=ab^2^/2, where a and b were tumor length and width, respectively (n=9 for each mutant group, mean ± SD). (C) Lysates prepared from the xenograft tumors harboring CTED mutants with or without dimerization impairing mutation were subjected to immunoblotting with antibodies against total or phospho-specific EGFR, Akt, and Stat3.

To investigate the requirement of asymmetric dimerization in tumorigenesis by CTED4, CTED5 and CTED8 mutants, NIH-3T3 cells stably expressing these mutants alone or in parallel with dimerization-impaired I941R or L704N *cis* mutants were xenografted into mice. The size of mouse tumors driven by CTED4, CTED5, or CTED8 mutants were significantly smaller in dimerization-disrupted L704N or I941R tumors (Fig. 5A and 5B). In line with these results, immunoblotting analysis revealed that constitutive phosphorylation of Akt and Stat3 was diminished in tumors driven by the dimerization-impaired CTED mutants (Fig. 5C).

Taken together with *in vivo* and *in vitro* results, we concluded that asymmetric dimerization of mutant receptor is required for oncogenic activation and consequent tumorigenesis by CTED mutants and that the acquired ability of forming constitutive dimerization, perhaps by C-terminal domain deletion, may be a key driving mechanisms of oncogenic activation of these variants.

## DISCUSSION

Our data provide mechanistic insights into the activation of 10 different C-terminal deletion variants including GBM and lung cancer-derived EGFR mutants. *In vitro* transformation experiments and *in vivo* xenograft studies indicate that constitutive asymmetric dimerization is a crucial mechanism of their oncogenic activation and consequent cellular transformation. We hypothesize that the ligand-independent asymmetric dimerization may be induced and/or favored by increased local concentration of mutant EGFR on the cell surface due to the impaired receptor internalization/degradation as shown in the previous studies [28, 37, 38], since C-terminal serves as a docking site for proteins involved in EGFR trafficking, such as CBL, MIG6, BRK/PTK6, and RAK [39]. In support of this hypothesis, we observed that the expression level of CTED mutants (CTED1 to CTED7) lacking autophosphorylation sites were higher than that of WT, CTED8, CTED9 and CTED10 mutants retaining Y1069, Y1092, and Y1110 residues that are known to either directly interact with Cbl sorting the EGFR to lysosome degradation, or indirectly via Grb2 to initiate ubiquitination upon activation [40]. These results are in agreement with previous reports showing that Grb2 does not co-precipitate in C-terminal truncated EGFR [33] and that C-terminal deletion mutants fail to internalize [37, 38, 41, 42]. However, our data do not exclude another possibility that truncated C-terminal domain of EGFR may directly or indirectly induce constitutive asymmetric dimerization in the absence of ligand by lowering the threshold of functional receptor dimerization [43-46].

Genomic characterization of cancers combined with chemical and immunological innovations have led to the development of multiple genome-guided targeted cancer therapies. The effective utilization and deployment of these therapies, however, is still in its infancy, as the interplay of mutation type, biochemical potency, oncogenic mechanism and clinical efficacy have not yet been fully elucidated. On the basis of our preclinical studies, we propose that a prospective targeted sequencing of *EGFR* C-terminal coding regions would be informative in genome-based patient selection for which administrating FDA-approved EGFR TKIs and cetuximab may be clinically beneficial. According to the recent large scale GBM genomic study [16], C-terminal rearrangements (namely CTED 2, 5, and 8) were detected at about 9% of GBM patients. However, since loss of C-terminus yields not mappable transcript, the authors postulate that EGFR CTED mutations are potentially more frequent than understood. Considering that we have provided pharmacologic evidence for uncharacterized CTED mutants as well, we think even larger proportion of patients with GBM or lung cancer may benefit from EGFR targeted therapies than what is predicted. Also, we speculate that combining antibodies and kinase inhibitors may bring synergistic effect in these patients, as dual EGFR inhibition has proven successful in several types of cancer [47].

Recently, we reported that dimerization-dependent L858R and G724S mutants were sensitive to cetuximab, whereas tumors driven by dimerization-independent mutants such as T790M were resistant, suggesting a close correlation between dimerization dependency and the pharmacological effects of cetuximab [30, 31]. Importantly, our finding that cetuximab is highly effective against dimerization-dependent CTED mutants, provides additional compelling evidence of our previously proposed hypothesis that disruption of dimerization may be among the antitumor mechanisms of cetuximab. Therefore, we propose that blockage of constitutive dimerization of C-terminal exonic deletion mutants with cetuximab may be an effective novel therapeutic strategy to treat a subset of cancer patients, along with patients harboring dimerization-dependent L858R and G724S oncogenic EGFR mutants.

Ligand-induced tyrosine phosphorylation on specific residues within C-terminal domain of EGFR is essential for mediating activation of signaling pathways and cellular transformation [48, 49]. However, previous reports have demonstrated that C-terminal truncated EGFR mutant retains signal transduction [22, 32, 50] and our functional and biochemical data also clearly demonstrate that C-terminal phosphorylation is not required for oncogenic transformation by mutant EGFR as mutant forms of EGFR were able to induce tumors in the absence of autophosphorylation. Consistent with our results, EGFR exon 19 deletion mutant was shown to retain its oncogenic activity in the absence of the C-terminal domain or autophosphorylation [33, 51]. Given that oncogenic CTED mutants including CTED1 to CTED7 were still able to activate the major signaling pathways including Akt and Stats in the absence of autophosphorylation in our *in vitro* models, we believe that Akt and/or Stats may play a crucial role in cellular transformation by these mutants. Interestingly, we observed that the level of constitutive phospho-Stat3, but not phospho-Akt, was dramatically decreased in the tumors that responded to treatment with all EGFR targeted drugs tested, suggesting that Stat3 may be a key signaling protein for C-terminal deletion mutant-driven tumorigenesis. This result coincide well with the report of Pines *et al* in which unsupervised examination of the phosphoproteome revealed that CTED8 activate Stat3 to a greater extent compared to WT-EGFR [22]. In addition, previous reports have demonstrated that C-terminal truncated mutants, but not WT EGFR, increase Src phosphorylation as well [22, 33]. Together with these data, our results lend support to the hypothesis that C-terminal truncated mutants may activate different downstream signaling pathways as they may have altered specificity for target proteins [50]. However, a detailed molecular mechanism underlying C-terminal phosphorylation-independent cellular transformation and downstream signaling activation by CTED mutants needs to be further characterized.

In addition, we observed that EGFR phosphorylation did not influence the oncogenic activity or EGFR targeted drug sensitivity, in which the IC_50_ values of different CTED mutants remained close in range irrespective of the presence or absence of autophosphorylation on the receptor. Also, we did not find any correlation between pharmacological effects with EGFR inhibitors and the length of C-terminal domain truncation in CTED mutants. In future studies, it will be informative to identify any common region or crucial motif within C-terminal domain responsible for oncogenic activation and/or drug sensitivity by more refined biochemical and functional approaches.

In summary, we demonstrated that truncation of EGFR C-terminal domain by intragenic deletion is a key oncogenic driving events in tumorigenesis. In addition, we showed that ligand-independent asymmetric dimerization is a potential mechanism for oncogenic transformation by these mutants. Finally, our findings will provide a rationale to develop a new class of drug to disrupt asymmetric dimerization, which would be potent to treat a subset of cancer patients harboring dimerization-dependent oncogenic EGFR mutants including C-terminal deletion mutants.

## MATERIALS AND METHODS

### Expression constructs

Various pBabe-puro expression constructs encoding CTED1 to CTED10 mutants (presented as schematic in Fig. 1A) were made by a series of recombinant PCR reactions. In brief, two separate PCR amplicons were generated using two pairs of primers designed to amplify the 5′ and 3′ region of sequences to be deleted using pBabe wild-type EGFR as a template. The resulting purified PCR fragments were annealed and amplified by the second round of PCR reaction. Then, the EGFR exonic deletion DNA fragments were subcloned into the pBabe wild-type EGFR plasmid vector, pre-cleaved with the restriction enzymes BglII and SalI. All plasmids were confirmed by sequencing.

### Cell culture and generation of cell lines by viral transduction

All EGFR CTED mutant expressing cell lines (Ba/F3 and NIH-3T3 cells) used in the study were established by retroviral infections, pooled and maintained as previously described [19, 30]. For EGFR stimulation experiments, cells were serum-starved for 18 hours followed by EGF (Biosource) treatment (50ng/mL) for 5 minutes before harvesting.

### Anchorage-independent growth assay in soft agar

Soft agar assays were performed in triplicate as previously described [52] with minor changes (3×10^4^ cells per well). Digital images were taken and the number of colonies were quantified after 3 weeks using GelCount software (Oxford) according to the manufacturer's protocol. The data represent triplicate wells. Each assay was repeated a minimum of two times with comparable results.

### Cell growth inhibition assay

For growth inhibition assays, Ba/F3 cells were seeded at a density of 1×10^4^ cells per well in 96-well plates (Corning). Twenty-four hours after plating, cells were treated with either cetuximab (MERCK Serono), erlotinib (LC laboratories), afatinib (LC laboratories), or dacomitinib (Selleckchem) at the indicated concentrations and incubated for another 72 hours. Viable cell numbers were measured using Cell Counting Kit-8 solution (Dojindo). Data are expressed as percentage of growth relative to that of untreated control cells.

### Immunoblotting and antibodies

Cells were lysed in RIPA buffer supplemented with protease inhibitors (Roche) and phosphatase inhibitors (Thermo Scientific). Whole-cell lysates were then separated using 6% or 8% SDS-PAGE followed by transferring to a nitrocellulose membrane. The membrane was probed with the antibodies against either N-terminal epitope EGFR (Thermo Scientific), C-terminal epitope EGFR (Bethyl), 4G10 (Millipore), β-actin, Stat3, p-Stat3, Akt, p-Akt, p-Stat5 or p-Shc (Cell Signaling Technology). Then, the blot was incubated overnight at 4°C and then with HRP-conjugated anti-rabbit or anti-mouse IgG antibody for 1 hour at room temperature. The proteins were then visualized with SuperSignal West Pico Chemiluminescent Substrate (Thermo Scientifc).

### Generation and treatment of xenografted mice

Animal experiments were carried out in accordance with IACUC of Laboratory Animal Research Center (LARC; AAALAC International-approved facility) in Samsung Medical Center. NIH-3T3 cells were resuspended in PBS and 1 to 2 million cells were injected subcutaneously into the nude mice, as previously described [19]. For antitumor effect of EGFR targeted therapies study, tumors were allowed to reach 50 to 70 mm^3^ in size, and then mice were randomly allocated to the control (PBS), cetuximab, erlotinib, or afatinib group. For the cetuximab-treated mice, we administered 1mg per mouse by intra-peritoneal injection 3 times per week, and for the afatinib and erlotinib-treated mice, we orally administered 1 mg per mouse 3 times per week. Tumor volume was measured 3 times per week using caliper and estimated from the equation V=ab^2^/2, where a and b were tumor length and width, respectively.

## SUPPLEMENTARY MATERIALS AND FIGURES


