# Supplementary material for: Constitutive asymmetric dimerization drives oncogenic activation of epidermal growth factor receptor carboxyl-terminal deletion mutants
Source: Oncotarget. 2015 Mar 12;6(11):8839–50. doi: 10.18632/oncotarget.3559 (PMC4496187; doi:10.18632/oncotarget.3559)
Supplement: Supplementary file 1 [file oncotarget-06-8839-s001.pdf]

Constitutive asymmetric dimerization drives oncogenic activation of epidermal growth factor receptor carboxyl-terminal deletion mutants

Supplementary Material

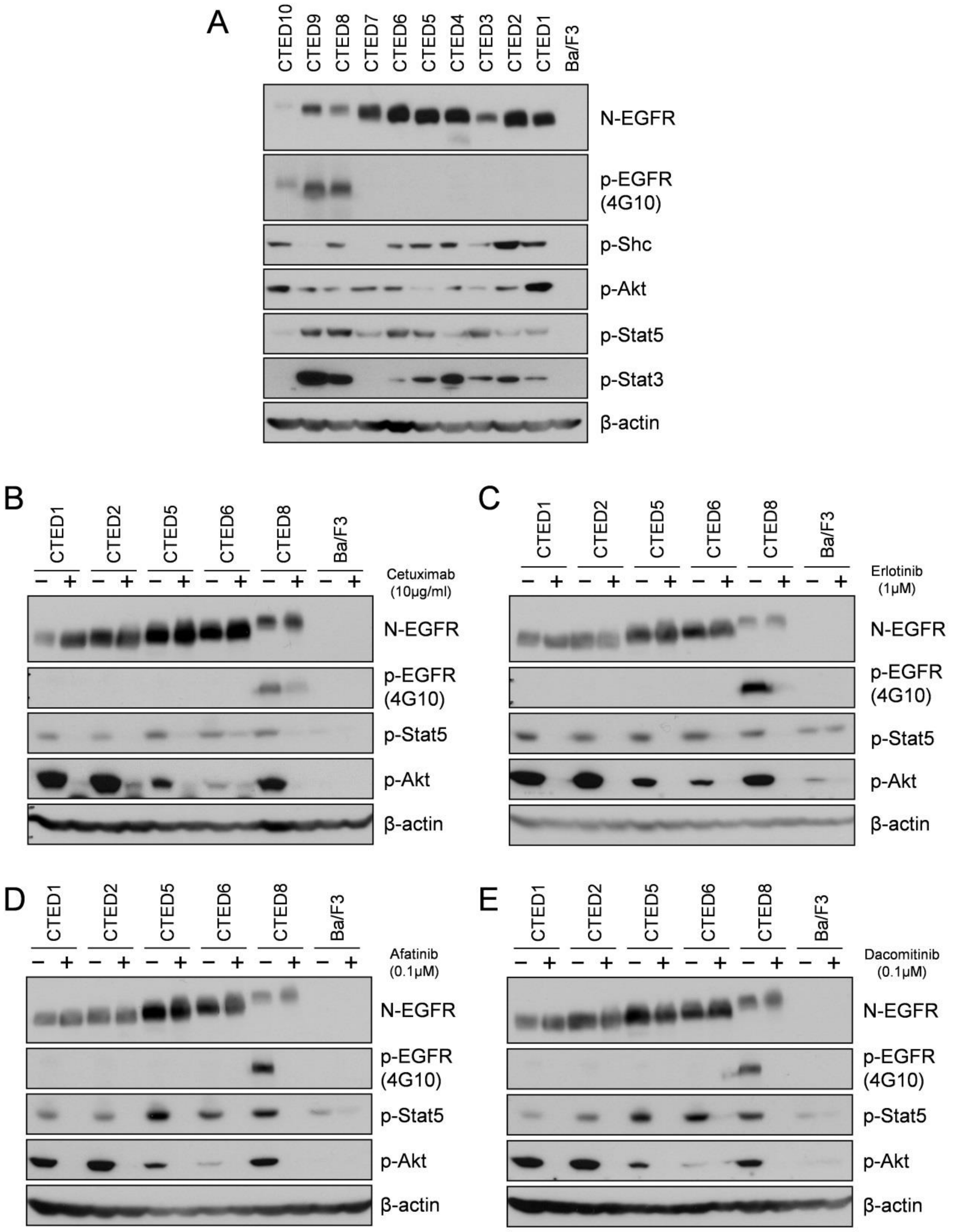

Supplementary Figure S1: Pharmacologic effects of cetuximab, erlotinib, afatinib, and dacomitinib against oncogenic CTED mutants *in vitro*.(A) Transformed Ba/F3 cells expressing the indicated EGFR CTED mutants were subjected to immunoblotting with phospho-tyrosine EGFR (4G10), phospho-Shc, phospho-Akt, phospho-Stat5, and phospho-Stat3 antibodies. Phosphorylation of EGFR, Stat5, and Akt were suppressed by either cetuximab **(B)**, erlotinib **(C)**, afatinib **(D)** or dacomitnib **(E)**.
